# Disability worsening among persons with multiple sclerosis and depression: A Swedish cohort study

**DOI:** 10.1212/WNL.0000000000008617

**Published:** 2019-12-10

**Authors:** Stefanie Binzer, Kyla A. McKay, Philip Brenner, Jan Hillert, Ali Manouchehrinia

**Affiliations:** From the Department of Clinical Neuroscience (S.B., K.A.M., J.H., A.M.), Department of Medicine Solna (P.B.), and Karolinska Neuroimmunology & Multiple Sclerosis Centre and Centre for Molecular Medicine (A.M.) Karolinska Institutet, Stockholm, Sweden, Odense University Hospital (S.B.), Department of Neurology, Denmark; and Karolinska University Hospital (J.H.), Stockholm, Sweden.

## Abstract

**Objective:**

Depression is common in multiple sclerosis (MS), but its impact on disability worsening has not yet been determined. We explored the risk of disability worsening associated with depression in a nationwide longitudinal cohort.

**Methods:**

This retrospective cohort study used linked data from 3 Swedish nationwide registries: the MS Register, National Patient Register, and Prescribed Drug Register. Two incident cohorts were developed: cohort 1 included all registered cases of MS in the MS Registry (2001–2014) with depression defined as ≥1 ICD-10 code for depression; and cohort 2 comprised all cases of MS in the MS Registry (2005–2014) with depression defined as ≥1 prescription filled for an antidepressant. Cox regression models were used to compare the risk of reaching sustained disability milestone scores of 3.0, 4.0, and 6.0 on the Expanded Disability Status Scale (EDSS) between persons with MS with and without depression.

**Results:**

Cohort 1 included 5,875 cases; 502 (8.5%) had depression. Cohort 2 had 3,817 cases; 1,289 (33.8%) were prescribed an antidepressant. Persons with depression were at a significantly higher risk of reaching sustained EDSS scores of 3.0, 4.0, and 6.0, with hazard ratios of 1.50 (95% confidence interval [CI] 1.20–1.87), 1.79 (95% CI 1.40–2.29), and 1.89 (95% CI 1.38–2.57), respectively. A similar increased risk among persons exposed to antidepressants was observed, with hazard ratios of 1.37 (95% CI 1.18–1.60), 1.93 (95% CI 1.61–2.31), and 1.86 (95% CI 1.45–2.40) for sustained EDSS scores of 3.0, 4.0, and 6.0, respectively.

**Conclusion:**

Persons with MS and comorbid depression had a significantly increased risk of disability worsening. This finding highlights the need for early recognition and appropriate treatment of depression in persons with MS.

Numerous studies have examined risk factors for the development of multiple sclerosis (MS),^1^ whereas more recently, long-term and population-based studies of factors affecting MS disability worsening have become feasible.^2^ Studies have shown that male sex, progressive disease course at onset, and older age at onset are associated with disability worsening.^3–5^ A paucity of evidence remains with regard to modifiable risk factors for MS disability worsening, but comorbid conditions are being increasingly explored as a potential avenue for intervention.^6,7^

The risk of depression is substantially increased in persons with MS and vice versa, even before the first MS symptom.^8,9^ A recent meta-analysis reported a pooled mean prevalence for depression in persons with MS of 30.5%.^10^ A Canadian cohort study showed that the incidence of depression was 71% higher in persons with MS.^11^ Psychiatric comorbid conditions greatly increase the risk of suicide in persons with MS^12^ and contribute to the socioeconomic burden of MS.^13,14^ Few longitudinal studies have examined the relationship between depression and MS disability worsening. A Canadian study found that persons with MS and a comorbid mood disorder had a higher Expanded Disability Status Scale (EDSS) score than patients with MS without a psychiatric comorbidity,^15^ while a Dutch study reported that baseline depression was not related to disability progression measured 10 years later.^16^ Thus, further studies are warranted; the aim of this study therefore was to examine the influence of comorbid depression or exposure to an antidepressant on the risk of MS disability worsening.

## Methods

### Data sources

Patients were identified from the Swedish MS register^17^ (SMSreg), a nationwide database established in 1996 that currently contains MS-specific information from all 64 neurology clinics in Sweden. SMSreg does not contain information on nonpharmacologic interventions. Individuals with a definite MS diagnosis who were registered in the SMSreg were linked to several population-based registers. The Swedish National Patient Register (NPR) contains information on all inpatient and outpatient specialized care (national coverage from January 1, 2001), coded according to the ICD-9/10 with a primary diagnosis and up to 30 secondary diagnoses. The NPR does not contain information on primary care (i.e., visits to a general practitioner). The Swedish Prescribed Drug Register (PDR) contains information on all prescriptions (including from primary care physicians) dispensed in pharmacies across Sweden, coded according to the Anatomic Therapeutic Classification (ATC) system from July 1, 2005. Data were available in all sources until December 31, 2014.

### Patient population

**Figure 1:**
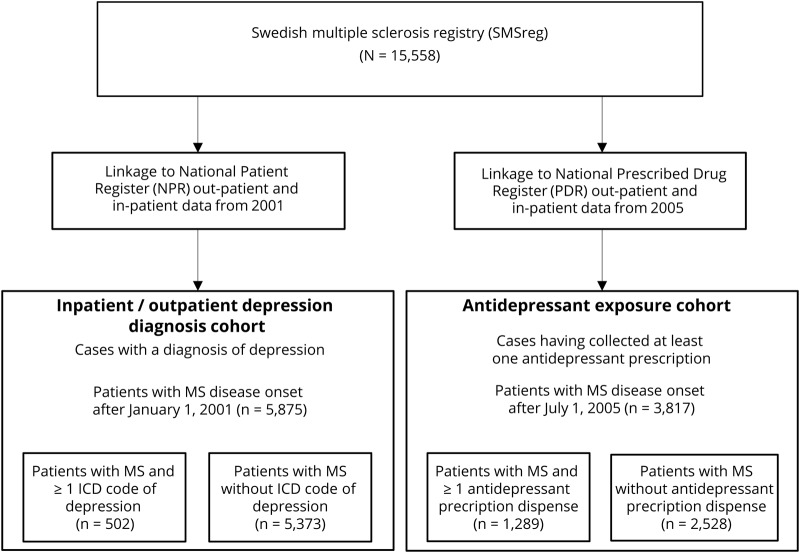
Flowchart of data collection in Swedish national registries MS = multiple sclerosis.

The study population included all individuals with a definite diagnosis of MS according to McDonald criteria^18^ and registered in the SMSreg. From these data, 2 cohorts were constructed (figure 1):

#### Cohort 1

Patients with ≥1 ICD-10 code for depression (F32–34) as a primary or secondary diagnosis in the NPR were categorized as depressed. Only persons whose date of MS onset (date of first manifestation of MS recorded by MS specialist neurologists) occurred on or after January 1, 2001, were included (to align with the start of complete outpatient NPR data availability). As a complementary analysis, we identified individuals with an ICD code for depression within 2 years before their MS disease onset to evaluate the effect of pre-MS depression on the risk of disability worsening. Patients with MS onset on or after January 1, 2003, were included in this analysis to ensure 2 years of pre-MS data availability.

#### Cohort 2

Patients with ≥1 prescription of antidepressant dispensed in the community were categorized as exposed to antidepressant. Antidepressants were identified from the PDR with the use of ATC codes. ATC is an international drug classification system maintained by the World Health Organization. The system classifies active substances in groups according to their therapeutic, pharmacologic, and chemical properties and the organ or system on which they act.^19^ We searched for the following antidepressant ATC codes: N06AA01 (desipramine), N06AA02 (imipramine), N06AA04(clomipramine), N06AA11 (protriptyline), N06AA12 (doxepin), N06AA17 (amoxapine), N06AA21 (maprotiline), N06AB03 (fluoxteine), N06AB04 (citalopram), N06AB05 (paroxetine), N06AB06 (sertraline), N06AB08 (fluvoxamine), N06AB10 (escitalopram), N06AF03 (phenelzine), N06AF04 (tranylcypromine), N06AG02 (moclobemide), N06AX06 (nefazodone), N06AX11 (mirtazepine), N06AX16 (venlafaxine), N06AX21 (duloxetine), N06AX23 (desvenlafaxine), N06AX12 (bupropion), N06AX22 (agomelatine), and N06AX26 (vortioxetine). Amitriptyline, nortriptyline, and buspirone were excluded because of their frequent off-label uses.^20^ Only persons whose MS onset occurred on or after July 1, 2005, were included (to align with the start of complete PDR data availability).

The 2 cohorts were not mutually exclusive; some patients both had an ICD code for depression and received antidepressant treatment. In each cohort, patients who did not have an ICD code for depression and had not received an antidepressant were labeled not classified with depression.

### Study outcomes

The study outcomes were risk of reaching sustained EDSS scores of 3.0 (moderate disability but no impairment of walking), 4.0 (significant disability but able to walk without aid or rest for 500 m), and 6.0 (requires unilateral assistance to walk about 100 m with or without resting) and conversion to secondary progressive MS (SPMS). EDSS scores were collected prospectively, and sustained EDSS score was defined such that all subsequent EDSS measurements must have been equal to or greater than the milestone of interest (EDSS score of 3.0, 4.0, or 6.0). Persons who met the milestone but had no subsequent EDSS recorded were censored at the last EDSS before they reached the milestone. Date of SPMS conversion was determined retrospectively by the treating neurologist according to the 1996 Lublin criteria.^21^

### Statistical analyses

Kaplan-Meier analysis was used to estimate the median time to reach disability milestones. Adjusted Cox proportional hazards regression models were used to calculate the risk of reaching disability milestones among persons with and without depression. All Cox models were controlled for potential confounders, including sex; age at MS onset; MS course at onset (relapsing-onset or primary progressive); physical comorbidity, defined by the Charlson Comorbidity Index^22^; and exposure to disease-modifying therapy (DMTs; as a time-varying covariate, such that persons could be categorized as treatment naive, but once they were exposed to a DMT, they were considered on drug for the remainder of follow-up). DMTs were categorized into first-line and second-line treatments. First-line treatments included interferon betas, glatiramer acetate, teriflunomide, and dimethyl fumarate. Second-line therapies included fingolimod, natalizumab, rituximab, and alemtuzumab. Depression, measured with an ICD code or an antidepressant dispensation, was also measured as time-varying, such that once a person met criteria for depression, he or she was considered to have it for the remainder of follow-up. Finally, a sex-stratified analysis of cohort 1 was explored.

#### Sensitivity analyses

Because we lacked primary care data, we anticipated the possibility of misclassification of persons with depression (who were treated by their primary care doctor and not as an inpatient or outpatient) as not classified with depression. We performed a sensitivity analysis to determine whether misclassification of patients would bias our estimates. To do so, we randomly assigned 5% and 20% of patients without depression to the depressed group and fit the models for the risk of reaching an EDSS score of 4.0.

We also performed a sensitivity analysis among cohort 1 in which we required ≥2 ICD codes of depression to be categorized as depressed.

All statistical analyses were performed with R: A Language and Environment for Statistical Computing, version 3.4.1 (R Foundation for Statistical Computing, Vienna, Austria; 2017).^23^

### Standard protocol approvals, registrations, and patient consents

This study was approved by the Stockholm regional ethics committee (EPN) at Karolinska Institutet. All patients provided informed consent for their health information to be used for research purposes.

### Data availability

Data related to the current article are available from Ali Manouchehrinia, Karolinska Institutet. To be able to share data, a data-transfer agreement needs to be completed between Karolinska Institutet and the institution requesting data access. This is in accordance with the data-protection legislation in Europe (General Data Protection Regulation). Persons interested in obtaining access to the data should contact Ali Manouchehrinia (ali.manouchehrinia@ki.se).

## Results

The table displays the number and characteristics of the 2 cohorts. Groups were similar with regard to sex distribution, age at MS onset, and exposure to first- and second-line treatments.

**Table:**
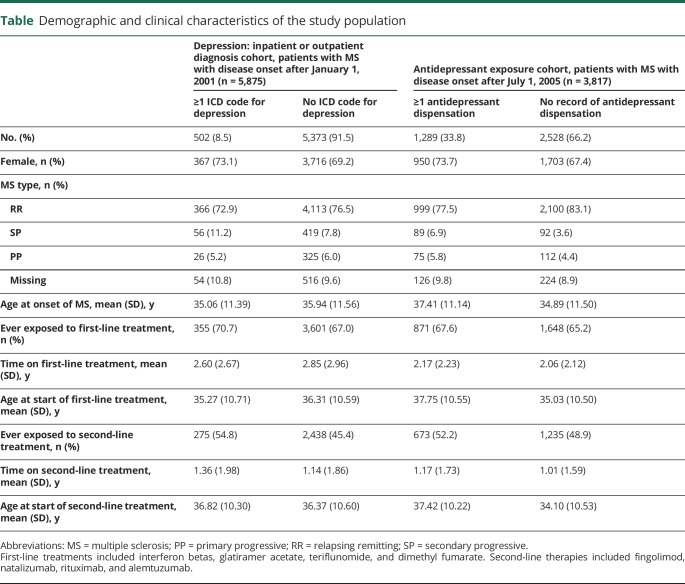
Demographic and clinical characteristics of the study population

**Figure 2:**
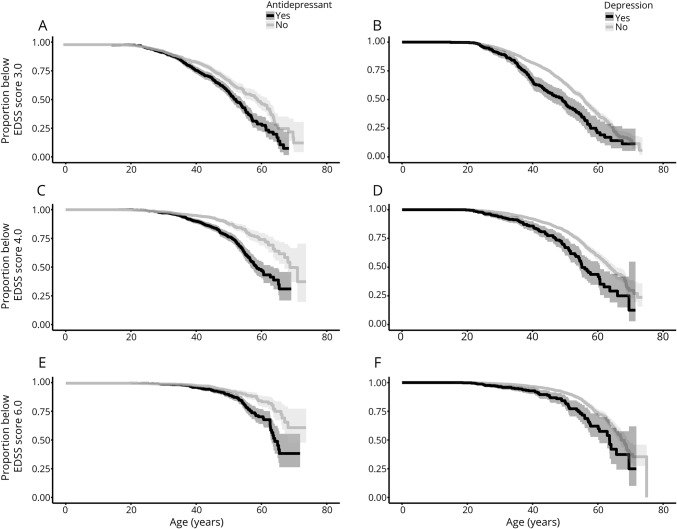
Kaplan-Meier estimates of age at EDSS score milestones Kaplan-Meier estimates of age at Expanded Disability Status Scale (EDSS) score milestones 3.0 (A and B), 4.0 (C and D), and 6.0 (E and F) stratified by depression diagnosis (A, C, and E) and exposure to antidepressants (B, D, and F).

Of 5,875 patients with MS onset between January 2001 and December 2014, 502 (8.5%) had at least 1 inpatient or outpatient visit for depression. These patients had an increased risk of reaching sustained EDSS milestones with hazard ratios (HRs) of 1.50 (95% confidence interval [CI] 1.20–1.87), 1.79 (95% CI 1.40–2.29), and 1.89 (95% CI 1.38–2.57) times of reaching sustained EDSS scores of 3.0, 4.0, and 6.0, respectively, relative to persons not classified with depression (figure 2). The risk of conversion to SPMS was not statistically significantly different between groups (HR 1.16, 95% CI 0.94–1.42). The relationship between depression and disability progression was significant among both men and women (sex-stratified results not shown). A higher Charlson Comorbidity Index also conferred a significant, although modest, increased risk of reaching EDSS scores of 3.0, 4.0, and 6.0 with an HR of 1.29 (95% CI 1.04–1.23), 1.08 (95% CI 1.03–1.23), and 1.09 (95% CI 1.04–1.13), respectively. The analysis of pre-MS onset depression, which included 175 patients with a diagnosis of depression before their first MS symptom, also revealed an increased risk of reaching EDSS scores of 3.0, 4.0, and 6.0 with adjusted HRs of 1.66 (95% CI 1.22–2.25), 2.27 (95% CI 1.54–3.35), and 2.25 (95% CI 1.37–3.70), respectively. The risk of conversion to SPMS was not significant (HR 1.33, 95% CI 0.66–2.70).

There were 3,817 patients with MS in cohort 2, 1,289 (33.8%) of whom had collected at least 1 prescription for an antidepressant between January 2005 and December 2014. Those exposed to antidepressants had a significantly higher risk of reaching sustained EDSS scores of 3.0, 4.0, and 6.0 compared to patients without depression with HRs of 1.37 (95% CI 1.18–1.60), 1.93 (95% CI 1.61–2.31), and 1.86 (95% CI 1.45–2.4), respectively (figure 2). The risk of conversion to SPMS did not reach significance (HR 1.22, 95% CI 0.84–1.77). There was an overlap of 281 persons between cohorts 1 and 2 who both had an ICD code for depression and received antidepressant treatment.

## Sensitivity analyses

On randomly assigning patients without depression to the depressed group, the risk remained significant (HR 1.68, 95% CI 1.38–2.04, *p* < 0.0001) when 5% of patients without depression were classified as depressed. When 20% of patients without depression were assumed to be depressed, the HR was 1.55 (95% CI 1.29–1.86, *p* < 0.0001).

When we applied more stringent criteria for the depression cohort, requiring ≥2 ICD codes for depression, the results remained significant with HRs of 1.50 (95% CI 1.13–2.00), 1.72 (95% CI 1.25–2.36), and 1.61 (95% CI 1.08–2.40) for EDSS scores of 3.0, 4.0, and 6.0, respectively.

## Discussion

We examined a nationwide cohort of patients with MS to assess the relationship between depression and MS disability worsening. Our results suggest that patients with MS with medically recognized depression, significant enough to warrant contact with specialist care or requiring treatment with an antidepressant, progress significantly faster than patients with MS without clinical indications of depression. This is also the case for those who are diagnosed with depression before their MS onset.

To the best of our knowledge, this is the largest study to examine this relationship to date. Our findings are concordant with a recent Canadian study that reported a higher EDSS score in persons with MS and comorbid depression; however, after stratification by sex, this relationship remained significant only among women.^15^ In the current study, we found a significant impact of depression on risk of disability worsening in both men and women. The inconsistent results may be explained by the stated lack of statistical power due to the small number of men in the Canadian study.

The observed association between depression and MS progression may be explained in a number of ways. First, depression could be a reaction to the experience of worsening of disability. For instance, feelings of hopelessness are more common in patients with SPMS than in patients with relapsing-remitting MS, suggesting that mood is, in part, a reaction to the course of disease.^24^ The observation that pre-MS depression was associated with increased risk of disability worsening suggests that this may not be the only explanation.

Second, patients with MS with depression are more likely to smoke^25^ and to be nonadherent to medication,^26^ each of which may contribute to a higher rate of disability worsening.^5,27^ People with depression are also less likely to exercise,^28^ and exercise has been shown to have beneficial effects in persons with MS such as improvements in brain volume.^29^ While we were able to account for the influence of physical comorbid conditions by adjusting for the Charlson Comorbidity Index, we lacked information on health behaviors, including exercise, smoking, and adherence to medication, which may have influenced the disability trajectory of patients. The age at exposure and length of exposure to DMTs in the cohorts with and without depression were similar. We cannot exclude the possibility that the increased disability worsening in persons with MS and comorbid depression may be partly due to DMT nonadherence. However, this relationship is not well established; some studies have found an association between depression and nonadherence in people with MS,^26^ while others reported no association.^30,31^

Third, pathogenic similarities may exist between CNS inflammation and depression that led to enhanced CNS vulnerability among these patients. While behavior and mood disorders have long been considered to result from biochemical alterations in neuronal networks, glia malfunctioning is now achieving greater attention.^32,33^ Currently, studies examining white matter tract damage resulting in disconnectivity between brain regions are being performed with regard to depressive symptoms in MS.^34–36^ A significant reduction in white matter integrity, a hallmark of MS, has been found in people with depression relative to healthy controls.^37^ Emotional changes have also been found to occur in the premotor symptomatic stage of experimental autoimmune encephalomyelitis mice.^38^

Our findings that antidepressant exposure led to a greater risk of disability led us to consider whether antidepressive treatment itself may contribute to disability worsening. We are unable to draw conclusions on the basis of the current study because we cannot separate the role of depression itself, the role of antidepressant treatment, and the potential for indication bias. Furthermore, this hypothesis does not align with the known properties of antidepressants, which include anti-inflammatory action^39^ and enhancement of neurogenesis.^40,41^

Strengths of this study include the use of population-based, nationwide registers of a large sample of patients. All of the information in the registers is collected prospectively, thereby reducing the possibility of recall bias. Important limitations of the study were the lack of access to primary care data and the lack of validation of depression with Swedish administrative data. This study examined depression in a specialist care setting, and the fact that the depression diagnosis was given by a specialist doctor strengthens the validity of the diagnosis. Furthermore, our sensitivity analysis requiring ≥2 ICD codes for depression to be categorized as depression did not significantly change our results.

We were unable to examine subgroups of depression (mild, moderate, severe) because ICD codes were only coded up to 3 levels, with no further subclassification. Furthermore, defining the degree of depression in a person can be challenging due to overreporting (ICD codes carried over between visits) and underreporting. Most persons in Sweden are treated for mental health in primary health care clinics, with more severe cases being admitted to hospital or seen in psychiatric outpatient clinics. Thus, there may be a risk of misclassification in that people with depression treated in primary care may be labeled as not diagnosed with depression. Furthermore, a stigma associated with depression still exists, which may hinder some from reporting their symptoms to a physician.^42^ Among the medical community, depression may be viewed as a symptom of MS, which may also lead to underreporting. Because we do not have access to primary care data, persons treated for depression exclusively in primary care would have been classified as nondepressed. However, our sensitivity analysis demonstrated that even with up to 20% misclassification of persons with depression as nondepressed, the relationship between depression and disability remained significant. Although antidepressants may be used on- or off-label in conditions other than depression, we were unable to explore this further because the indication for the prescription is rarely included in the PDR.

Another important limitation was the inability to make a distinction between chronic and nonchronic or situational depression. By analyzing depression as a time-varying covariate, we were able to measure a person’s trajectory of disease progression after medical recognition of depression or antidepressant use. However, we were unable to assess whether the depression had ameliorated and whether this effected disability.

Clearly, effective management of depression in persons with MS is needed. First, the identification of depression among persons with MS must be improved.^6^ Validated depression screening tools exist for the MS population that can be implemented in the clinical setting.^43^ The appropriate approach will differ from patient to patient but could include pharmacologic and/or nonpharmacologic interventions such as cognitive behavioral therapy and exercise.^44^

Patients with MS with depression are at a significantly increased risk of disability worsening. The causal mechanisms of these associations are not yet established but may be both biological and psychosocial. Regardless of the underlying reason, our observations emphasize that depression is an important comorbidity to be recognized and appropriately managed in patients with MS. It may be used in risk stratification and aid in better providing personalized medicine for persons with MS. Future studies should examine whether effective treatment to reduce the burden of depression in MS has the potential to minimize MS disability.

## Glossary

ATC: Anatomic Therapeutic Classification
CI: confidence interval
DMT: disease-modifying therapy
EDSS: Expanded Disability Status Scale
HR: hazard ratio
ICD-9/10: *International Classification of Diseases, 9th/10th revision*
MS: multiple sclerosis
NPR: National Patient Register
PDR: Prescribed Drug Register
SMSreg: Swedish MS register
SPMS: secondary progressive multiple sclerosis
